# Strain- and Sex-Dependent Circadian Changes in Abcc2 Transporter Expression: Implications for Irinotecan Chronotolerance in Mouse Ileum

**DOI:** 10.1371/journal.pone.0020393

**Published:** 2011-06-03

**Authors:** Alper Okyar, Enza Piccolo, Constance Ahowesso, Elisabeth Filipski, Virginie Hossard, Catherine Guettier, Rosanna La Sorda, Nicola Tinari, Stefano Iacobelli, Francis Lévi

**Affiliations:** 1 INSERM, U776 Rythmes Biologiques et Cancers, Hôpital Paul Brousse, Villejuif, France; 2 Université Paris-Sud, UMR-S0776, Orsay, France; 3 Assistance Publique-Hôpitaux de Paris, Unité de Chronothérapie, Département de Cancérologie, Hôpital Paul Brousse, Villejuif, France; 4 Department of Pharmacology, Istanbul University Faculty of Pharmacy, Beyazit, Istanbul, Turkey; 5 Consorzio Interuniversitario Nazionale per la Bioncologia (CINBO) CE.S.I. - Università “G. d'Annunzio”, Chieti, Italy; 6 Assistance Publique-Hôpitaux de Paris, Laboratoire d'Anatomie et Cytologie Pathologiques, Hôpital Paul Brousse, Villejuif, France; Vanderbilt University, United States of America

## Abstract

**Background:**

ATP-binding cassette transporter abcc2 is involved in the cellular efflux of irinotecan. The drug is toxic for mouse ileum, where abcc2 is highly expressed. Here, we investigate whether circadian changes in local abcc2 expression participate in the circadian rhythm of irinotecan toxicity for ileum mucosa, and further assess whether genetic background or sex modify this relation.

**Methodology/Principal Findings:**

Ileum mucosa was obtained every 3–4 h for 24 h in male and female B6D2F_1_ and B6CBAF_1_ mice synchronized with light from Zeitgeber Time (ZT)0 to ZT12 alternating with 12 h of darkness. Irinotecan (50 mg/kg i.v. daily for 4 days) was administered at the sex- and strain-specific times corresponding to least (ZT11-15) or largest drug-induced body weight loss (ZT23-03-07). *Abcc2* expression was determined with qRT-PCR for mRNA and with immunohistochemistry and confocal microscopy for protein. Histopathologic lesions were graded in ileum tissues obtained 2, 4 or 6 days after treatment. Two- to six-fold circadian changes were demonstrated for mRNA and protein mean expressions of *abcc2* in mouse ileum (p<0.05). ZT12 corresponded to high mRNA and protein expressions, with circadian waveforms differing according to genetic background and sex. The proportion of mice spared from ileum lesions varied three-fold according to irinotecan timing, with best tolerability at ZT11-15 (p = 0.00003). Irinotecan was also best tolerated in males (p = 0.05) and in B6CBAF_1_ (p = 0.0006).

**Conclusions/Significance:**

Strain- and sex-dependent circadian patterns in abcc2 expressions displayed robust relations with the chronotolerance of ileum mucosa for irinotecan. This finding has strong potential implications for improving the intestinal tolerability of anticancer drugs through circadian delivery.

## Introduction

Molecular circadian clocks rhythmically control Phase I and II metabolism over the 24 hours in mammals [1]–[3]. The molecular clocks in each cell are coordinated through an array of physiological rhythms generated by the suprachiasmatic nuclei (SCN) in the hypothalamus. Such circadian timing system determines rhythmic changes in drugs effects [3]. This is particularly relevant for anticancer drugs, with a narrow therapeutic index. Indeed, circadian timing modifies 2- to 10-times the extent of toxic effects of 40 anticancer medications in rodents, and up to 5-fold that of 5-fluorouracil-oxaliplatin in cancer patients [4]–[6]. The mechanisms of drug detoxification involve the cellular efflux of medications and/or their metabolites via specific transporters. However the possible relevance of a circadian regulation of drug transporters for anticancer drug tolerability has not been addressed yet.

Many anticancer drugs and/or their metabolites are expelled out of cells by ATP-binding cassette (ABC) transporter proteins which function as ATP-dependent molecule pumps in the cell membrane. ABCC2 (human)/abcc2 (rodent) is one such ABC transporter also known as multidrug resistance-associated protein-2 (MRP2/mrp2) or canalicular multispecific organic anion transporter (cMOAT).

ABCC2/abcc2 overexpression confers multidrug resistance (MDR) to colon, breast, lung or renal cell carcinomas [7], [8]. ABCC2/abcc2 is also expressed in healthy tissues, where it could influence the toxic effects of anticancer agents, especially for the gastrointestinal tract. The highest mRNA expression of *abcc2* was found in liver for rats and humans, and in small intestine for mice, with a decreasing gradient from duodenum to ileum [9]–[11]. Lowest expression was found in mouse colon [10]. Abcc2 protein expression in rat ileum was localized in the brush-border membrane, and with highest values in the apical part of the villus rather than in the crypt [12].

The gut toxicities of irinotecan (7-ethyl-10-[4-(1-piperidino)-1-piperidino]-carbonyloxycamptothecin) limit the use of this effective drug against gastro-intestinal malignancies [13]. Large and consistent circadian changes in irinotecan toxicities were found in mice whose circadian timing system was synchronized with an alternation of 12 h of light from ZT0 to ZT12 (zeitgeber time) and darkness from ZT12 to ZT24/0. Best tolerability corresponded to drug dosing between ZT7 and ZT15 according to strain, sex and experimental conditions [14], [15]. In an initial study in male B6D2F_1_, severe ileum mucosa lesions were identified in mice receiving irinotecan at ZT19 to ZT3, but not in those treated at ZT7 to ZT15. Thus, best ileum tolerability corresponded to irinotecan dosing between mid-rest and early activity span [15]. In subsequent investigation, irinotecan toxicity at ZT7 was least in female as compared with male mice, and varied significantly according to mouse strain. Furthermore, both genetic background and sex significantly altered the molecular prediction of irinotecan toxicity by the mRNA expression of enzymes involved in drug bioactivation, such as carboxylesterase 2 (*ces2*), detoxification, such as UDP-glucuronosyltransferase (*ugt1a1*), and DNA replication, such as topoisomerase 1 (*top1*) [16].

Irinotecan detoxification also involves its cellular efflux through abcc2 [17]. This transporter is known to display circadian changes in liver and intestine [18], [19]. Here, we specifically investigated the relations between abcc2 expression and irinotecan toxicity in mouse ileum. Circadian rhythms are shown at mRNA and protein expression levels through quantitative RT-PCR and confocal immunohistochemistry respectively. These results are qualified with regard to villus localization, strain and sex.

The study is the first one to demonstrate that abcc2 mRNA and protein expressions are regulated along the 24 h in mouse ileum mucosa. Circadian rhythm in abcc2 expression contributes to that in irinotecan tolerability. We further discuss its relevance for tolerability pattern of other anticancer drug effluxed by abcc2 as well.

## Materials and Methods

### Animals and synchronization

The study was conducted in accordance with the guidelines approved for animal experimental procedures by the French Ethical Committee (decree 87–848). All the experiments involved male and female B6D2F_1_ or B6CBAF_1_ mice 7 weeks of age purchased from Janvier (Le Genest St Isle, France). They were synchronized with an alternation of 12 h of light and 12 h of darkness (LD12∶12) in an autonomous chronobiological facility (Jouan-Thermo Electron LED S.A.S., Saint-Herblain, France) with food and water *ad libitum* for 3 weeks prior to any intervention. Each facility is equipped with temperature control (23±1°C) and comprises four or six compartments, each one being provided with separate filtered air (100±10 l/min) and lighting regimen. Light intensity at cage level ranged from 223 to 315 lux. All the manipulations during the dark phase were performed under dim red light (7 lux).

### Experimental design

Circadian rhythms in the mRNA expression of *abcc2* were first investigated separately in ileum serosa and in scraped ileum mucosa of 30 male B6D2F_1_ mice sampled at one of six ZT equispaced by 4 h (Study 1). Time (in hours) was referred to ZT0 (light onset).

In order to enhance temporal resolution, subsequent experiments involved sampling of scraped ileum mucosa at one of eight ZT separated by 3 h, in 40 male and 40 female B6D2F_1_ (♀C57BL/6×♂DBA/2), or 40 male and 40 female B6CBAF_1_ mice (♀C57BL/6×♂CBA) for the determination of mRNA expression patterns as a function of ZT, gender and genetic background (Study 2).

In Study 3, the abcc2 protein localization was assessed using immunohistochemistry, confocal microscopy and quantification through image analysis at 4 circadian times associated with high (ZT12 and ZT15) or low (ZT0 and ZT3) mRNA expressions, as defined in study 2. Study 3 involved 20 male or female mice of both strains.

Study 4 tested the hypothesis that ileum toxicity of irinotecan would differ as a function of circadian timing, genetic background and sex in a total of 66 mice. Irinotecan was administered at a circadian time previously shown to determine “best” or “worst” tolerability, based on body weight loss in each mouse group [16].

### RNA extraction

Total RNA from liver was purified using the method of Chomczynski and Sacchi [20] and stored at −80°C until use. PCR was performed with a LightCycler 480 (Roche, Meylan, France) using SYBR green I dye detection according to the manufacturer's recommendations. cDNA was added to a reaction mixture (Faststart DNA SYBR Green I; Roche Diagnostics, Meylan, France) with appropriate primers at 0.5 µM each. Relative mRNA abundance was calculated using a standard curve method. Expression levels were normalized to the levels of the constitutively and non-rhythmically expressed *36B4* (acidic ribosomal phosphoprotein P0). The following primers were used for *abcc2* accession number: NM-013806.2; forward, 5′-GCTGAGATCGGAGAGAAGGGTA- 3′ and reverse, 5′-CACTTGGGGAAGGAAGTGAA -3′, for *36B4* Accession number: NM_007475.3; forward 5′-GCTGATGGGCAAGAACACCA-3′ and reverse 5′-CCCAAAGCCTGGAAGAAGGA-3′.

### Protein expression studies

Mouse ileum was fixed in 4% buffered formalin at room temperature for 24 h. The fixed tissues were then dehydrated in ethanol, cleared in Bioclear (Bioptica, Milan, Italy) then embedded into paraffin. Sections were cut to a thickness of 5 µm and transferred to positively charged slides. Slides were incubated overnight at 37°C and immersed in xylol in order to remove paraffin. Tissue sections were rehydrated in a graded alcohol series (100%, 96% and 80%) for 5 min in each bath and rinsed in running tap water. Haematoxylin and Eosin (H&E) staining was performed according to a routine protocol in order to estimate the quality of the tissue. Immunohistochemistry analysis was performed using a tissue microarray with cylindrical cores of 2 mm of paraffin-embedded tissue [21].

### Immunohistochemistry and confocal microscopy

A microwave (three times five minutes, at 600 W) method using citrate buffer (pH = 6) was used for antigen retrieval. Sections were incubated for 60 min at room temperature with Rodent Block M (Biocare Medical, Concord, CA) and then incubated for 30 min with 1% BSA in PBS to minimize nonspecific binding. A rabbit anti-mouse abcc2 antibody (LifeSpan Technologies, Seattle, USA) diluted 1∶25 in PBS, or a mouse anti-mouse β-catenin antibody (Santa Cruz Biotechnology, Santa Cruz, CA) diluted 1∶50 in PBS were then incubated over night at 4°C. After three washes in PBS, sections were co incubated for 60 min at room temperature with Alexa Fluor 488-conjugated goat anti-rabbit secondary antibody or Alexa Fluor 546-conjugated goat anti-mouse from Molecular Probes (Invitrogen, Milan, Italy) and with the far-red fluorescent DNA dye DRAQ5 (Alexis, Lausen, Switzerland) to visualize nuclei. As a negative control, tissues were stained with rabbit IgG (Jackson Laboratories, Bar Harbor, Maine) at the same concentrations used for the primary antibody. Following three brief PBS washes, samples were mounted using an aqueous mountant, Eukitt (Bioptica, Milan, Italy).

### Image analysis

Images were acquired with the Zeiss LSM 510 meta-confocal microscope using a 488, 546 and 633-nm lasers. Detector gain voltages and pinhole were set at the beginning of the experiment and maintained constant during the acquisition of all samples. For each tissue section, three images were collected corresponding to different areas of the section. To provide a quantitative estimate of abcc2 protein expression, images were analyzed with Image ProPlus 6.0 software (Media Cybernetics, Bethesda, MD). The standard version of the software was modified in order to measure the fluorescent area in an automatic manner (Immagini&Computer, Milan, Italy). Briefly, at the beginning of the acquisition analysis, the fluorescence background was subtracted from each image; then the software estimated the number of nuclei and measured the fluorescent area. The obtained value was then divided by the number of nuclei to obtain a mean value of fluorescence area. To reduce inter-assay variability, mean values were expressed as a percentage of abcc2 expression in a control ileum from a BALB/c mouse.

### Drug

The clinical solution for intravenous (i.v.) injection of irinotecan (20 mg/mL; Aventis, Milan, Italy) was diluted in 0.9% sodium chloride on each study day, prior to injections. The final drug solution was administered i.v. at the dose of 50 mg/kg into the right retro-orbital venous sinus once daily for 4 consecutive days [15], [16]. Irinotecan was injected at the ZT corresponding to best tolerability of irinotecan, i.e. ZT11 in male B6D2F_1_ and ZT15 in female B6D2F_1_ and in male or female B6CBAF_1_ mice, or to worst tolerability, i.e. ZT23 in male B6D2F_1_, ZT3 in female B6D2F_1_, and ZT7 in male or female B6CBAF_1_ according to prior study [15], [16].

### Histologic lesions

Ileum toxicities were assessed in 6–8 mice of each group treated at each ZT two, four and six days after irinotecan treatment. Sampling was performed at the same ZT as that of drug administration. Three control animals were also used per sampling time point and per group. The ileum was obtained immediately after sacrifice and fixed into 4% formaldehyde. Twenty four hours later, the samples were dehydrated and embedded into paraffin. Sections were made and stained with hemalun-erythrosine-safran. Each slide was examined by the same histopathologist and lesions were graded in a blind manner. The occurrence of ileum lesions located in surface epithelial cells, villi structure and/or crypt gland cells were scored as “1” for each of them. The sum of all three scores was computed as being a toxicity grade, theoretically ranging from 0 (normal) to 3 (alteration in each item).

### Statistical analyses

Means and SEM were calculated for each studied variable. Differences of mRNA expression of *abcc2* according to ZT, genetic background and sex were validated using multiple-way analyses of variance (ANOVA). The statistical significance of a sinusoidal 24 h rhythm was further documented by Cosinor [22]. This method further computed the parameters of the best-fitting 24-h cosine function with their 95% confidence limits, i.e. mesor (mean), amplitude (half the extent of variation) and acrophase (time of maximum, referred to ZT0). Intergroup differences in protein expression and ileum lesion scores were statistically validated by multiple-way ANOVA. Incidence data were compared by χ^2^ test. Rhythm parameter comparison tests involved Hotelling t-test [23]. All statistical tests were two-sided and performed using SPSS v.16 for Windows software (Paris-La Défense, France).

## Results

### Focal and circadian mRNA expression in ileum of male B6D2F_1_

**Figure 1:**
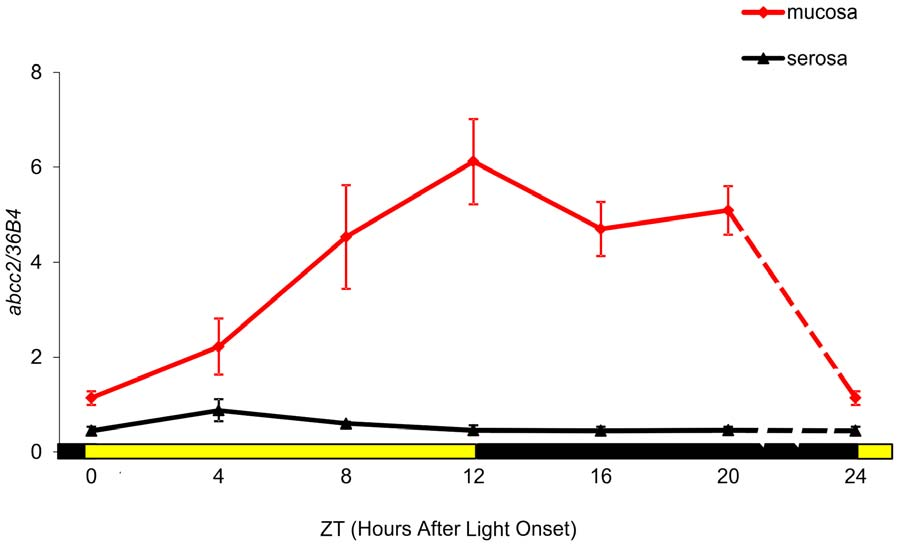
Circadian patterns of a*bcc2* mRNA expression in the ileum of male B6D2F_1_ mice. Mean (± SEM) in mucosa and in serosa as a function of Zeitgeber Time (ZT), with ZT0 as light onset. Statistically significant differences according to ZT were validated for mucosa with ANOVA (p = 0.02). A statistically significant 24 h rhythm was confirmed for mucosa with cosinor (p = 0.0011).

Mean mRNA expression of *abcc2* in ileum mucosa varied three-fold over the 24 h in male B6D2F_1_ mice, according to results from Study 1. Highest values were found in the first half of the dark span, with a peak at ZT12, while the nadir occurred at ZT0 (ANOVA, p = 0.02) (Figure 1). A sinusoidal pattern in *abcc2* expression was further confirmed with cosinor (p = 0.0011). The circadian amplitude was 55% of the mesor (95% Confidence Limits, CL: 30 to 80), and the acrophase was located at ZT13∶35 (11∶50 to 15∶25). Conversely, there was barely any mRNA expression in ileum serosa (Figure 1), without any significant rhythm detection (cosinor, p = 0.07).

### Relevance of strain and gender for the circadian expression of *abcc2* transcription in ileum mucosa

**Figure 2:**
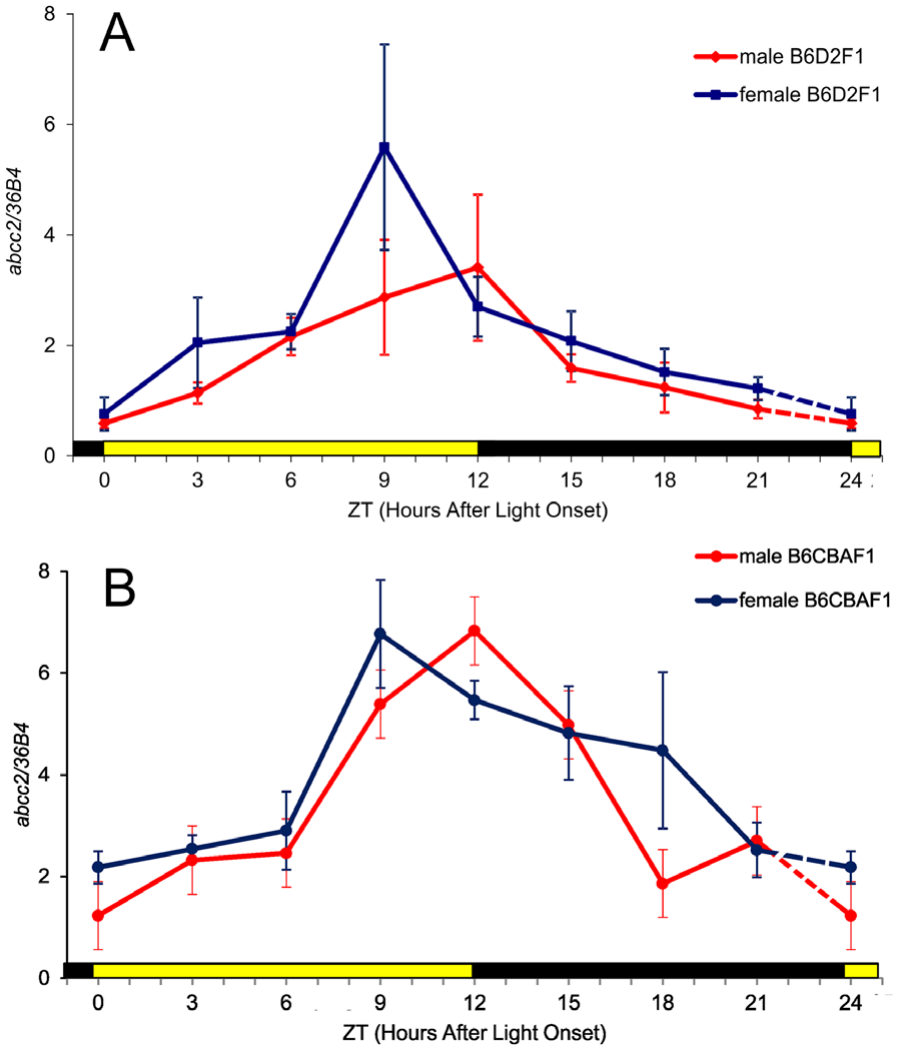
Circadian patterns of a*bcc2* mRNA expression in the ileum mucosa of mice, according to genetic background and sex. Mean (± SEM) as a function of Zeitgeber Time (ZT), with ZT0 as light onset. (A) male and female B6D2F_1_; (B) male and female B6CBAF_1_. Mean *abcc2* expression increased six-fold from ZT0 (trough) to ZT12 (peak) for B6D2F_1_ males and ZT0 (trough) to ZT9 (peak) for B6D2F_1_ females (ANOVA, p = 0.04; Cosinor, p = 0.0023 for B6D2F_1_ males and ANOVA, p = 0.0008; Cosinor, p = 0.0023 for B6D2F_1_ females). Similar three- to four-fold circadian variations were found in male and female B6CBAF_1_, with highest values occurring from ZT9 to ZT15, and a trough at ZT0 (ANOVA, p = 0.004 in male; p = 0.003 in female). The rhythm was validated by cosinor for B6CBAF_1_ (p = 0.00026 and p = 0.00012 in male and female, respectively).

In study 2, mean mRNA expression of *abcc2* also varied six-fold over the 24 h, with a peak at ZT12 and a nadir at ZT0 in male B6D2F_1_ (ANOVA, p = 0.04) (Figure 2A), Cosinor analysis further confirmed a sinusoidal pattern (p = 0.0023). The circadian amplitude was 72% of the mesor (35 to 110), and the acrophase was localized at ZT10∶20 (8∶20 to 12∶25). Thus, both studies 1 and 2 demonstrated a circadian rhythm with similar characteristics for the *abcc2* mRNA expression in the ileum mucosa of male B6D2F_1_ mice.

The mRNA expression of *abcc2* also displayed a statistically significant six-fold variation over the 24 h in the ileum mucosa of female B6D2F_1_ (ANOVA, p = 0.008). While the nadir occurred at ZT0, the peak time was located at ZT9 (Figure 2A). The circadian amplitude was 70% of the mesor (33 to 106) and the acrophase occurred at ZT 9∶30 (7∶25 to 11∶40) as estimated by cosinor (p = 0.0023).

Similar three- to four-fold circadian variations were found in male and female B6CBAF_1_, with highest values occurring from ZT9 to ZT15, and a trough at ZT0 (Figure 2B) (ANOVA, p = 0.004 in male; p = 0.003 in female). The sinusoidal pattern of these rhythms was confirmed by cosinor for mice of each sex (p = 0.00026 and p = 0.00012, respectively). The circadian parameters were similar in males and females, with respective relative amplitude of 68% of the mesor [38 to 98] and 51% [30 to 73] – and respective acrophases occurring at ZT11∶40 (9∶55 to 13∶20) and ZT11∶50 (10∶05 to 13∶30). Three-way ANOVA of all the mRNA expression data of *abcc2* revealed statistically significant differences as a function of circadian time (p<0.001) and genetic background (p<0.001), but not sex (p = 0.106). However, the comparison of circadian acrophases showed statistically significant differences between males and females B6D2F_1_ (p = 0.009), but not between males and females B6CBAF_1_ (p = 0.77).

### Relevance of strain and sex for the circadian expression of abcc2 protein in ileum mucosa (Study-3)

**Figure 3:**
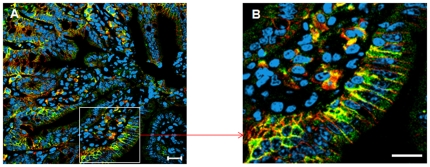
Co-localization of abcc2 and β-catenin in the membrane of ileum mucosa cells. Double immunofluorescence staining of abcc2 (green) and β-catenin (red) in mouse ileum using confocal laser scanning microscopy. Far-red fluorescent DNA dye DRAQ5 (blue) for visualizing nuclei. Co-localization between abcc2 and β-catenin was shown as yellow. A) Original magnification 40× (scale bar, 20 µm). B) Higher magnification of selected area.

**Figure 4:**
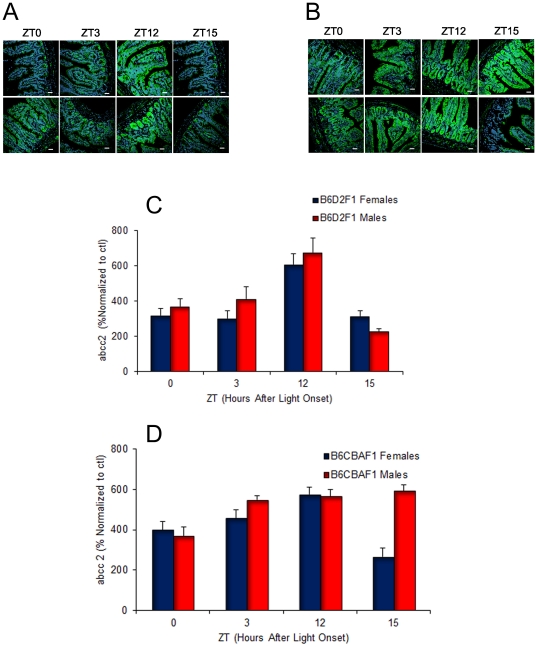
Confocal immunohistochemistry imaging of abcc2 protein expression in mouse ileum, according to circadian time, genetic background and sex. A) Selected examples in male (upper row) and female (lower row) B6D2F_1_. B) *Id* in B6CBAF_1_. C) Histogram depicting the changes in mean (±SEM) protein expression at four ZTs associated with low or high mRNA expressions in male and female B6D2F_1_. D) *Id* in B6CBAF_1_. NOTE: Three-way ANOVA confirmed statistically significant differences as a function of ZT (p<0.001), strain (p = 0.048). Statistically significant interactions were detected between ZT*strain (p = 0.001), ZT*sex (p = 0.035) and ZT*strain*sex (p = 0.004).

Abcc2 protein was mostly expressed in the plasma membrane and occasionally in the nuclear membrane of the cells in ileum mucosa, as showed by abcc2 co-localization with a known plasma membrane marker, likeβcatenin (Figure 3A). The spatial distribution patterns were similar whatever the sex or the strain (Figures 4A and 4B). Protein expression was barely detectable in the colon, so that no quantification was attempted. The expression of abcc2 protein displayed consistent 24 h changes in male and female B6D2F_1_, as well as in male and female B6CBAF_1_, with apparent strain- and/or sex-dependent differences. Protein expression peaked at ZT12 both in male and female B6D2F_1_ while it was lowest at ZT15 in males and at ZT15, ZT0 and ZT3 in females (Figure 4C). The extent of circadian variation was ∼three-fold in males and ∼two-fold in females. In B6CBAF_1_, abcc2 protein expression was highest from ZT12 to ZT15 in males and at ZT12 in females. Lowest values were found at ZT0 in males and at ZT15 in females (Figure 4D). The range of change was ∼two fold in female mice and much weaker in males. Three-way ANOVA validated statistically significant differences as a function of ZT (p<0.001) and strain (p = 0.048), but not sex (p = 0.07). Furthermore, statistically significant interactions were found between ZT and strain (p = 0.001), and between ZT and sex (p = 0.035) as well as all three factors (p = 0.004. Overall, mean protein expression was correlated with mean mRNA expression in each group at each ZT (r = 0.52; p<0.05).

### Relevance of strain and sex for ileum chronotolerance for irinotecan (Study-4)

**Figure 5:**
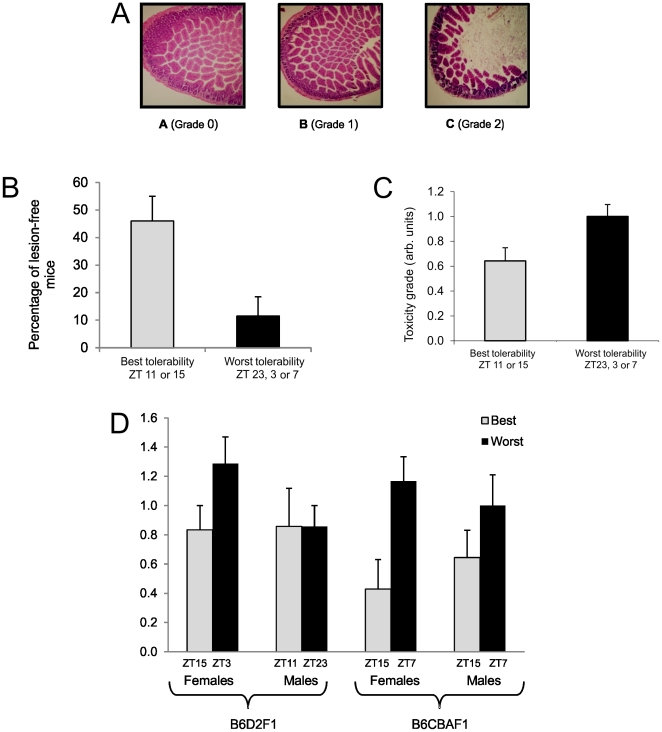
Tolerance of mouse small intestine for irinotecan, as a function of circadian timing, genetic background and sex. According to previous studies, the circadian timing (as ZT) corresponding to best tolerability of irinotecan was ZT11 in male B6D2F_1_ and ZT15 in female B6D2F_1_ as well as in male or female B6CBAF_1_ mice. The worst tolerability resulted from irinotecan dosing at ZT23 in male B6D2F_1_, ZT3 in female B6D2F_1_, and ZT7 in male or female B6CBAF_1_. Transverse sections of ileum 2 days after the fourth daily dose of irinotecan (50 mg/kg/d×4 days). Hemalun-erythrosine-safran staining. A, control, Grade 0; B, Grade 1; C, Grade 2. Bar graph of the proportion (±SEM) of mice free of ileum lesions (Grade 0) according to circadian timing of irinotecan (χ^2^, p = 0.00003). Bar graph of the proportion (± SEM) of toxicity grade according to circadian timing of irinotecan (ANOVA, p = 0.016). (D) Histogram of mean (± SEM) grade of ileum toxicity according to circadian timing, genetic background.and sex. The incidence of mice without any ileum damage was significantly higher in male as compared to female mice, irrespective of strain (χ^2^, p = 0.05), and in B6CBAF_1_ as compared to B6D2F_1_ (χ^2^, p = 0.0006). Three-way ANOVA validated the relevance of “best” vs. “worst” circadian timing (p = 0.018).

Histologic lesions in ileum were found in mice from each strain and sex, with scores up to two (Figure 5A). Ileum mucosa was mostly damaged two days after treatment completion, with full recovery within four days in all groups. Irinotecan timing determined the incidence of mice free of ileum lesions (grade 0) as well as the average lesion score. Thus no ileum damage was observed in 46.4% of the mice receiving irinotecan at the ZT predicted to achieve best tolerability as compared to 11.5% of those treated at the ZT predicted to yield poorest tolerability (13/28 vs. 3/26; p fromχ^2^ = 0.00003) (Figure 5B). The respective mean scores were 0.64±0.11 and 1.00±0.01 for the respective “best” and “worst” times (p from ANOVA = 0.016) (Figure 5C).

The incidence of mice without any ileum damage was ∼twice as high in male as compared to female mice, irrespective of strain (5/26, 19.2% vs 9/28, 32%; p from χ^2^ = 0.05), and in B6CBAF_1_ as compared to B6D2F_1_ (10/27, 37% vs 4/27, 14.8%; p from χ^2^ = 0.0006). Three-way ANOVA further confirmed the relevance of “best” vs. “worst” circadian timing (p = 0.018), with possible effects of strain (p = 0.06) and timing*sex interaction (p = 0.07). Thus, Figure 5D illustrates that circadian timing played a prominent role for ileum tolerability in all groups except male B6D2F_1_.

## Discussion

The current study is first to demonstrate that *abcc2/*abcc2 expression is strongly regulated along the 24 h in mouse ileum mucosa. The magnitude of the circadian changes ranged from 3- to 6-fold for mRNA expression and from 2- to 3-fold for protein expression. Accordingly, the ileum tolerability of irinotecan, an anticancer drug whose cellular efflux involves abcc2, was improved nearly fourfold with drug dosing near the time of maximum *abcc2*/abcc2 expressions.

The multiple circadian controls of drug absorption, distribution, metabolism, elimination and toxicity (ADMET) partly account for dosing time dependencies in the pharmacokinetics and toxic effects of medications [3], [6]. Indeed, circadian changes were shown for irinotecan disposition and tolerability in mice [14], [15]. Limited human data suggested that this was also the case in cancer patients [6]. Circadian clocks control the transcription of some ABC family members, including abcc2 (mrp2) in mouse liver [18], [24] and intestine [19], [25], [26]. Since irinotecan efflux is actively mediated by abcc2, and this gene is highly expressed in ileum mucosa, we hypothesized that abcc2 rhythms in ileum would participate in small intestine chronotolerance for irinotecan [15].

Abcc2 was expressed weakly and without any rhythm in ileum serosa as shown in this study, as well as in colon mucosa [9], [10]. On the contrary, both mRNA and protein expressions of abcc2 were high and rhythmic in the apical region of the small intestine, where the efflux function of abcc2 involves substrate carrier from apical to basolateral side [7]. While the organ specificity of clock-controlled genes was previously shown [27]–[29], our study further emphasized that rhythmic gene expressions could also depend upon the functional role of a specific area within a given organ. This implies that a better knowledge of the functional relevance of circadian rhythms should stem from careful examination of different regions in the same organ. The circadian amplitudes of the mRNA expression of *abcc2* or other ABC transporters in mouse or rat small intestine were low in the studies involving total ileum segment and large in that of ileum mucosa [19], [26], [30]. Maximum expression in total jejunum was reported to occur at ZT8 in male C57BL/6J mice and at ZT12 in male Sprague-Dawley rats [19], [30] i.e. in the second half of the rest span of these nocturnally-active rodents.

The rhythmic patterns in *abcc2* expression were similar in male and female B6CBAF_1_, while they differed between male and female B6D2F_1_, with regard to both amplitude and phase. In addition, mean *abcc2* expression was nearly doubled in B6CBAF_1_, as compared to B6D2F_1_. Thus, strain- and sex- related differences characterized the circadian mRNA expression of *abcc2*. They were confirmed for the protein expression of this gene, at four selected ZT, located near maximum and minimum mRNA expressions. While both mRNA and protein expressions peaked near ZT12 in male and female mice from either strain, the temporal patterns varied largely as a function of genetic background and sex. Consistently with the mRNA data, mean protein expression was also highest in B6CBAF_1_ as compared to B6D2F_1_.

Irinotecan damaged ileum mucosa over the two days following treatment completion, while full recovery was noticed two days later, in good agreement with prior report [15]. The tolerability of mouse ileum mucosa for irinotecan was largely improved following drug administration at ZT11 or ZT15, pending upon genetic background and sex. Furthermore, overall tolerability was best in male mice, and in B6CBAF_1_.

Thus, the maxima in circadian mRNA and protein expressions of abcc2 in ileum mucosa occurred near ZT12, when irinotecan tolerance was best in pooled groups of mice. However, wrongly timed irinotecan produced less ileum toxicity in male B6D2F_1_ than in male B6CBAF_1_ or in females from any strain. Irinotecan also damaged other target tissues such as colon and bone marrow. Lesions in colon mucosa were prominent in male but not in female B6D2F_1_ mice. The main toxicity in female B6D2F_1_ was hematologic rather than colic [31]. Multiple mechanisms are involved in irinotecan chronotolerance at many tissue levels, including liver, ileum and colon mucosa as well as bone marrow, with strain and sex dependencies. This was shown for liver mRNA expressions of *ces2*, *ugt1a1* and *top1*, three enzymes respectively involved into the bioactivation of irinotecan, its detoxification and its molecular toxicity [16]. Current and previous data support highest detoxification - high ileum abcc2 and liver ugt1a1 mRNA and protein expressions – and lowest bioactivation and target availability- low expressions of ces2 and top1, respectively in B6CBAF_1_ as compare to B6D2F_1_. Indeed, ileum toxicity was least in B6CBAF_1_ as compared to B6D2F_1_. These results at tissue toxicity level were consistent with those earlier found for body weight loss [16]. As a consequence, the relevance of the circadian pattern in abcc2 mRNA and protein expressions in ileum has to be interpreted against the several other determinants of irinotecan chronotolerance such as ugt1a1, ces2, top1 rhythms in different tissues.

In aggregate, our study shows that the local circadian control of ABCC2/abcc2-mediated detoxification strongly depends upon tissue location, genetic background and sex. This detoxification rhythm can importantly contribute to host tolerability for irinotecan and probably other anticancer drugs such as topotecan, cisplatin, docetaxel, doxorubicin, epirubicin, methotrexate, paclitaxel, and vinblastine whose efflux involves abcc2 [7]. Interestingly, a circadian rhythm has been shown for the tolerability of each of these agents in mice or rats [6]. However, several mechanisms jointly account for the chronopharmacology of anticancer drugs at cellular, tissue and whole organism level. This statement calls for the integration of our finding into a comprehensive systems biology approach to cancer chronotherapeutics [32]–[34]. The challenge is to identify the theoretically optimal chronotherapeutic schedules that will best spare healthy tissues from toxic insults in an individual patient.

## References

[1] Gachon F, Olela FF, Schaad O, Descombes P, Schibler U (2006). The circadian PAR-domain basic leucine zipper transcription factors DBP, TEF, and HLF modulate basal and inducible xenobiotic detoxification.. Cell Metab.

[2] Yang X, Downes M, Yu R, Bookout A, Weimin H (2006). Nuclear receptor expression links the circadian clock to metabolism.. Cell.

[3] Lévi F, Schibler U (2007). Circadian rhythms: mechanisms and therapeutic implications.. Annu Rev Pharmacol Toxicol.

[4] Lévi F, Zidani R, Misset JL (1997). Randomised multicentre trial of chronotherapy with oxaliplatin, fluorouracil, and folinic acid in metastatic colorectal cancer. International Organization for Cancer Chronotherapy.. Lancet.

[5] Lévi F, Focan C, Karaboue A, de la Valette V, Focan-Henrard D (2007). Implications of circadian clocks for the rhythmic delivery of cancer therapeutics.. Adv Drug Deliv Rev.

[6] Lévi F, Okyar A, Dulong S, Innominato PF, Clairambault J (2010). Circadian timing in cancer treatment.. Annu Rev Pharmacol Toxicol.

[7] Jemnitz K, Heredi-Szabo K, Janossy J, Ioja E, Vereczkey L (2010). ABCC2/Abcc2: a multispecific transporter with dominant excretory functions.. Drug Metab Rev.

[8] Szakács G, Paterson JK, Ludwig JA, Booth-Genthe C, Gottesmann MM (2006). Targeting multidrug resistance in cancer.. Nat Rev Drug Discov.

[9] Gotoh Y, Suzuki H, Kinoshita S, Hirohashi T, Kato Y (2000). Involvement of an organic anion transporter (canalicular multispecific organic anion transporter/multidrug resistance-associated protein 2) in gastrointestinal secretion of glutathione conjugates in rats.. J Pharmacol Exp Ther.

[10] Maher JM, Slitt AL, Cherrington NJ, Xingguo C (2005). Tissue distribution and hepatic and renal ontogeny of the multidrug resistance-associated protein (MRP) family in mice.. Drug Metab Dispos.

[11] Berggren S, Gall C, Wollnitz N, Ekelund M, Karlbom U (2006). Gene and protein expression of P-Glycoprotein, MRP1, MRP2, and CYP3A4 in the small and large human intestine.. Mol Pharm.

[12] Mottino AD, Hoffman T, Jennes L, Vore M (2000). Expression and localization of multidrug resistant protein mrp2 in rat small intestine.. J Pharmacol Exp Ther.

[13] Kweekel D, Guchelaar H, Gelderblom H (2008). Clinical and pharmacogenetic factors associated with irinotecan toxicity.. Cancer Treat Rev.

[14] Ohdo S, Makinosumi T, Ishizaki T, Yukawa E, Higuchi S (1997). Cell cycle-dependent chronotoxicity of irinotecan hydrochloride in mice.. J Pharmacol Exp Ther.

[15] Filipski E, Lemaigre G, Liu XH, Mery-Mignard D, Mahjoubi M (2004). Circadian rhythm of irinotecan tolerability in mice.. Chronobiol Int.

[16] Ahowesso C, Piccolo E, Li XM, Dulong S, Hossard V (2010). Relations between strain and gender dependencies of irinotecan toxicity and UGT1A1, CES2 and TOP1 expressions in mice.. Toxicol Lett.

[17] Innocenti F, Kroetz DL, Schuetz E, Dolan E, Ramírez J (2009). Comprehensive pharmacogenetic analysis of irinotecan neutropenia and pharmacokinetics.. J Clin Oncol.

[18] Panda S, Antoch MP, Miller BH, Su AL, Schook AB (2002). Coordinated transcription of key pathways in the mouse by the circadian clock.. Cell.

[19] Ando H, Yanagihara H, Sugimoto K, Hayashi Y, Tsuruoka S (2005). Daily rhythms of P-glycoprotein expression in mice.. Chronobiol Int.

[20] Chomczynski P, Sacchi N (2006). The single-step method of RNA isolation by acid guanidinium thiocyanate–phenol–chloroform extraction: twenty-something years on.. Nat Protoc.

[21] Kononen J, Bubendorf L, Kallioniemi A, Bärlund M, Schraml P (1998). Tissue microarrays for high-throughput molecular profiling of tumor specimens.. Nat Med.

[22] De Prins J, Hecquet B, Touitou Y, Haus E (1992). Data processing in chronobiological studies.. Biological rhythms in clinical and laboratory medicine.

[23] Bingham C, Arbogast B, Guillaume GC, Lee JK, Halberg F (1982). Inferential statistical methods for estimating and comparing cosinor parameters.. Chronobiologia.

[24] Zhang YK, Yeager RL, Klaassen CD (2009). Circadian expression profiles of drug-processing genes and transcription factors in mouse liver.. Drug Metab Dispos.

[25] Claudel T, Cretenet G, Saumet A, Gachon F (2007). Crosstalk between xenobiotics metabolism and circadian clock.. FEBS Lett.

[26] Murakami Y, Higashi Y, Matsunaga N, Koyanagi S, Ohdo S (2008). Circadian clock-controlled intestinal expression of the multidrug-resistance gene mdr1a in mice.. Gastroenterology.

[27] Storch KF, Lipan O, Leykin I, Viswanathan N, Davis FC (2002). Extensive and divergent circadian gene expression in liver and heart.. Nature.

[28] Miller BH, McDearmon EL, Panda S, Hayes KR, Zhang J (2007). Circadian and CLOCK-controlled regulation of the mouse transcriptome and cell proliferation.. Proc Natl Acad Sci USA.

[29] Ukai H, Ueda HR (2010). Systems biology of mammalian circadian clocks.. Annu Rev Physiol.

[30] Stearns AT, Balakrishnan A, Rhoads DB, Ashley SW, Tavakkolizadeh A (2008). Diurnal rhythmicity in the transcription of jejunal drug transporters.. J Pharmacol Sci.

[31] Ahowesso C (2010). Preclinical model for the personalization of cancer chronotherapeutics..

[32] Clairambault J (2009). Modelling physiological and pharmacological control on cell proliferation to optimise cancer treatments..

[33] Altinok A, Lévi F, Goldbeter A (2009). Identifying mechanisms of chronotolerance and chronoefficacy for the anticancer drugs 5-fluorouracil and oxaliplatin by computational modeling.. Eur J Pharm Sci.

[34] Lévi F, Altinok A, Goldbeter A, Cesario A, Marcus FB (2010). Circadian rhythms and cancer chronotherapeutics.. Cancer Systems Biology, Bioinformatics and Medicine: Research and Clinical Applications.

